# PIK3CA mutations confer resistance to first-line chemotherapy in colorectal cancer

**DOI:** 10.1038/s41419-018-0776-6

**Published:** 2018-07-03

**Authors:** Qiang Wang, Yan-long Shi, Kai Zhou, Li-li Wang, Ze-xuan Yan, Yu-lin Liu, Li-li Xu, Shi-wei Zhao, Hui-li Chu, Ting-ting Shi, Qing-hua Ma, Jingwang Bi

**Affiliations:** 1 Department of Oncology, General Hospital of Jinan Military Command PLA, Jinan, 250031 China; 2 Department of Pathology, 401 People’s Liberation Army Hospital, Qingdao, 266071 China; 3 Institute of Pathology and Southwest Cancer Center, Southwest Hospital, Third Military Medical University (Army Medical University), Chongqing, 400038 China; 4 Department of Oncology, Liaocheng Cancer Hospital, Liaocheng, 252000 China

## Abstract

Chemotherapy represents an important treatment option for colorectal cancer (CRC), but only half of the patients benefit from these regimens. We explored the potential predicting value and mechanism of PIK3CA mutation in CRC chemotherapy. CRC specimens from 440 patients were retrospectively collected and examined with a fluorescence PCR-based method. The correlation of first-line chemotherapy response and PIK3CA mutation was evaluated according to follow-up and medical records. The underlying mechanism of PIK3CA mutation in chemotherapy resistance was assessed with CRC tumors and primary cells. The mutation frequency of the *PIK3CA* gene in CRC patients was 9.55%, which was correlated with late TNM staging and lower histological grade. The CRC patients with *PIK3A* mutation showed worse response to first-line chemotherapy than those without *PIK3CA* mutation. PIK3A mutation tumor cells showed poor sensitivity to first-line chemotherapy in vitro and in vivo. PIK3CA mutation induced PI3K/Akt signaling activation to increase LGR5^+^ CRC stem cells survival and proliferation, from which lead to chemotherapy resistance. Furthermore, *PIK3CA* ^mutation^/LGR5^+^ expression was an independent detrimental factor for CRC patients. Our findings indicated that PIK3CA mutation induced PI3K/Akt activation contributed to CRC stem cells survival and proliferation, from which cells further resistance to chemotherapy. PIK3CA ^mutation^/LGR5^+^ expression was a potential biomarker for monitoring chemotherapy resistance in CRC.

## Introduction

As one of the most prevalent malignancies, colorectal cancer (CRC) displays an increasing incidence in the past decades, which ranks the forth common cancer worldwide and the second leading cause of cancer-related death nowadays^1^. Systemic chemotherapy is still the mainstay treatment besides surgery and local radiotherapy. The first-line chemotherapy, such as FOLFOX and FOLFORI^2^, was widely used in advanced CRC, but chemotherapy resistance occurred in a large number of patients^3,4^. The biomarkers for predicting and monitoring chemotherapy resistance were valuable in such first-line regimens application^5,6^.

Phosphatidylinositol-4,5-bisphosphate 3-kinase, catalytic subunit alpha (PIK3CA) is one of the most commonly mutated genes in CRC^7–9^. *PIK3CA* encodes the p110 catalytic subunit of PI3K, which is one of the crucial kinases in PI3K/Akt/mTOR signaling^10,11^. *PIK3CA* mutation hot spots are located at five sites in exons 9 and 20^12^, which activate PI3K/AKT signaling pathway to contribute to carcinogenesis, cellular growth, proliferation and survival of multiple solid tumors^13–18^. However, it’s still controversial of the correlation between PIK3CA mutations and prognosis of CRC patients^19,20^.

The impact of PIK3CA mutations on first-line chemotherapy regimens remains unclear. This retrospective study evaluated the chemotherapy response and *PIK3CA* mutations in patients with CRC in our hospital, as well as determined the potential role of PIK3CA mutations in chemotherapy resistance.

## Results

### Correlation of *PIK3CA* mutations with clinicopathologic characteristics of CRC patients

Table 1 showed the baseline characteristics of the 440 CRC patients. The median age of the cohort was 59 years (range from 24 to 86 years). Male patients account for 61.59% (271/440), whereas 38.41% of female patients (169/440). Totally, 253 (57.50%) patients suffered from rectal cancer and 187 patients (42.50%) had colon cancer, including left-sided colorectal cancer (LCRC) in 104 patients (23.64%) and right-sided colon cancer (RCC) in 83 patients (18.86%). Histologically poor differentiated tumor was observed in 100 patients (22.73%), and lymph node metastasis was observed in 165 patients (37.50%). Totally, 203 patients (46.14%) were diagnosed as advanced CRC (TNM stage III–IV). The frequency of *PIK3CA* gene mutations in CRC patients was 9.55% (42/440). The mutated loci were mainly located on exon 9 (29/42, 69.05%), including E545K 50% (21/42), E542K 11.90% (5/42) and E545D 7.14% (3/42), respectively. Another 13 CRC patients had mutations on exon 20 (30.95%). The frequencies for H1047R and H1047L were 28.57% (12/42) and 2.38% (1/42), respectively.

**Table 1:** Baseline patient characteristics of the 440 colorectal patients

Characteristic	Number of patients (%)
Gender	
Male	271 (61.59)
Female	169 (38.41)
Median age (range), years	59 (24–86)
ECOG performance status	
0	398 (90.45)
1, 2	42 (9.55)
Primary tumor location	
RCC	83 (18.86)
LCRC	104 (23.64)
Rectal	253 (57.50)
Histological grade	
Low	100 (22.73)
Middle-high	340 (77.27)
LN metastasis	
Yes	165 (37.50)
No	275 (62.50)
TNM staging	
I–II	237 (53.86)
III–IV	203(46.14)
PIK3CA status	
Wild-type	398 (90.45)
Mutant	42 (9.55)
Chemotherapy regimen	
FOLFOX or XELOX	421 (95.68)
FOLFIRI	19 (4.32)

RCC was defined as a tumor arising from the cecum to the transverse colon, excluding the appendix, while LCRC was defined as a tumor arising from the descending colon to the rectum

*RCC* cecum to the transverse colon, *LCRC* descending to the rectosigmoid colon and rectum

The correlation between *PIK3CA* mutation and clinicopathologic characteristics was analyzed in Table 2. Significant correlations were observed in *PIK3CA* mutation with histological grade and TNM stage (*p* < 0.001 and *p* = 0.034, respectively). *PIK3CA* mutation in tumor tissues was correlated with lower histological grade and late clinical stage. However, no significant correlation of *PIK3CA* mutation was shown with age, gender, ECOG scores, tumor location and lymph node metastasis (*p* > 0.05, respectively).

**Table 2:** Correlation of *PIK3CA* mutations with clinicopathological characteristics of CRC patients

Characteristic	Wild type (%)	Mutant (%)	*P* value
Total	398	42	
Gender			
Male	242	29	0.296
Female	156	13	
ECOG			
0	359	39	0.577
1, 2	39	3	
Age			
≤59	198	22	0.746
>59	200	20	
Tumor location			
RCC	76	7	0.702
LCRC	322	35	
Histological grade			
Low	80	20	<0.001
Medium-High	318	22	
LN metastasis			
Yes	152	13	0.357
No	246	29	
TNM stage			
I–II	220	17	0.034
III–IV	178	25	

### *PIK3CA* mutation correlates with first-line chemotherapy resistance

**Fig. 1:**
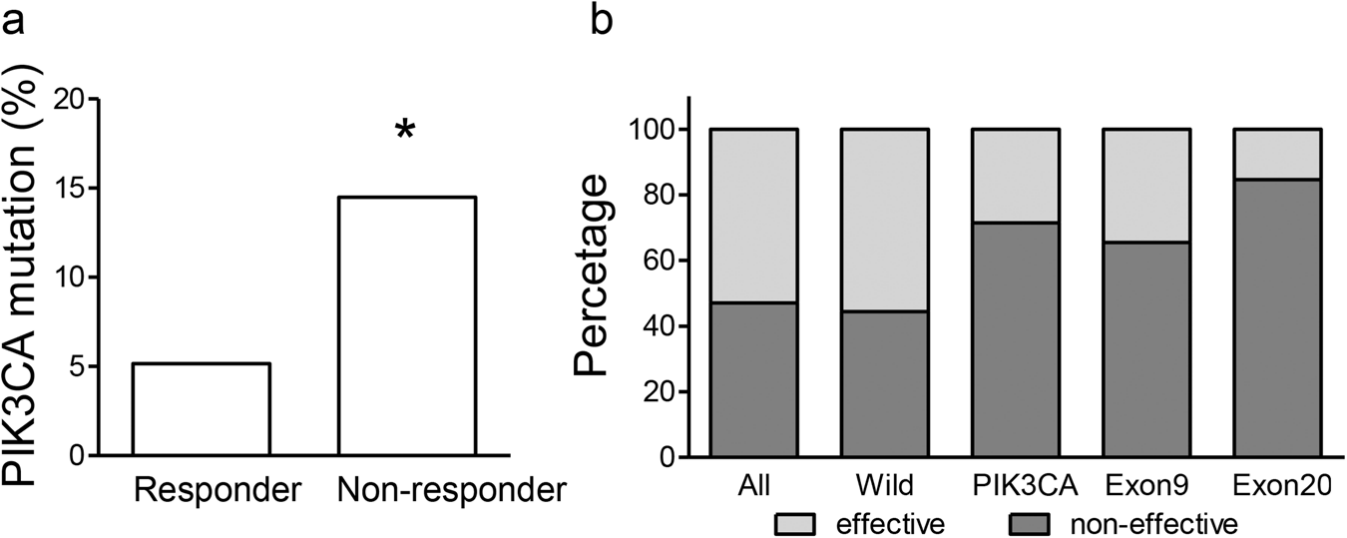
The correlation between chemotherapy response and *PIK3CA* mutation status in CRC patients. **a** The percentage of *PIK3CA* mutation in the patients effective and non-effective to first-line chemotherapy. *p < 0.05. **b** Chemotherapy rsponses among patients with wild-type, *PIK3CA* mutant, exon 9 mutant and exon 20 mutant tumors

All patients involved in this study received first-line chemotherapy regimens, which were FOLFOX and XELOX in 420 cases (95.68%, Table 1). Disease progression was observed in 47.05% (207/420) patients within 3 years after surgery, which was defined as non-effective to chemotherapy treatments. The patients resistant to first-line chemotherapy showed higher percentage of *PIK3CA* mutation than those effective (14.49 vs. 5.15%, *p* = 0.003. Fig. 1a). The patients with *PIK3CA* mutant tumor showed higher non-effective rate than the others (71.43 vs. 44.47%. Fig. 1b and Table 3). Furthermore, the patients with *PIK3CA* exon 9/20 wild-type and corresponding mutant-type tumors also showed significant differences in disease-progression rates (19.78%, *p* = 0.031; 38.71%, *p* = 0.006, respectively. Fig. 1b and Table 3). Meanwhile, elevated disease-progression rate in the patients with *PIK3CA* mutant tumors than whole cohort (24.38%, *p* = 0.003, Fig. 1b), which indicated a poor chemotherapy response in PIK3CA mutant CRC.

**Table 3:** Chemotherapy response according to *PIK3CA* mutation status

	*PIK3CA* wt vs. mt	*PIK3CA*-exon9 wt vs. mt	*PIK3CA*-exon20 wt vs. mt
Effective (*n*)	221 vs. 12	223 vs. 10	231 vs. 2
Non-effective (*n*)	177 vs. 30	188 vs. 19	196 vs. 11
Progression (%)	44.47 vs. 71.43	45.74 vs. 65.52	45.90 vs. 84.62
Difference	26.96	19.78	38.71
*P* value^a^	0.001	0.031	0.006

*wt* wild-type, *mt* mutant

^a^Calculated using Fisher’s exact test

### PIK3CA mutation leads to chemotherapy resistance of CRC primary cells

**Fig. 2:**
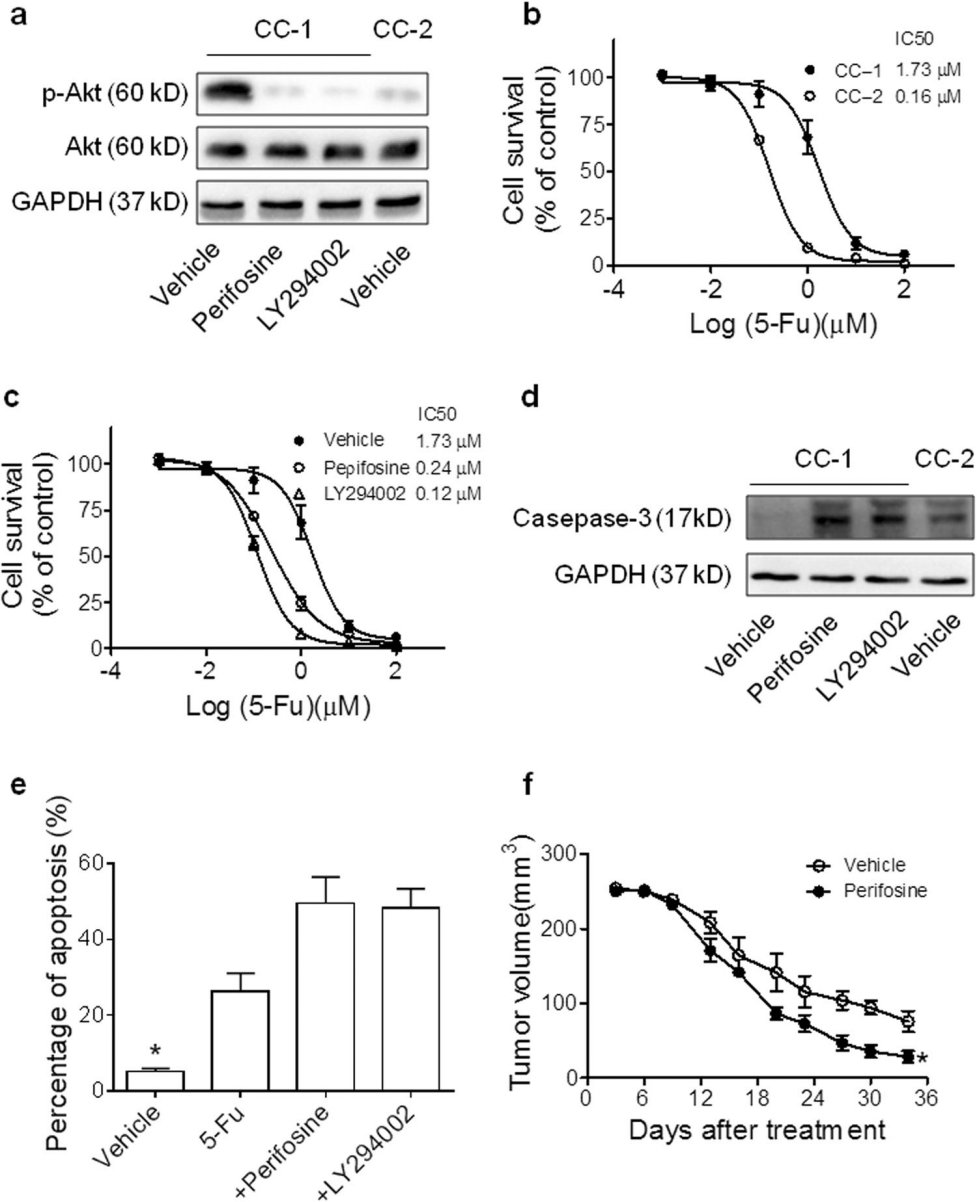
PIK3CA mutation correlates with first-line chemotherapy resistance. **a** Western blot analysis of pAkt, Akt and GAPDH (loading control) in primary CRC cells. **b** CC-1 and CC-2 cells were incubated with 5-Fu (25 µM) for 72 h. Cell viability was examined by the MTT assay. Results represent the mean ± S.E.M. of the values relative to vehicle controls; *P < 0.05. **c** CC-1 cells were incubated combined perifosine or LY294002 with 5-FU. Cell viability was examined by the MTT assay. The data are shown as means ± S.E.M. **d** Immunoblots showing caspase-3 and GAPDH (loading control) expression levels in CC-1 cells treated with perifosine or LY294002 combined with 5-Fu (1 μM) for 24 h, and in CC-2 cells. **e** The colorectal cancer cells (CC-1) were treated with perifosine, LY294002 or 5-Fu for 48 h and the cells were collected for apoptosis analysis by flow cytometry (*n* = 3). **f** In vivo tumorigenicity of CRC xenograft tumors treated with or without perifosine. The tumor growth was monitored every week until 6 weeks after injection. Data are shown as means ± S.E.M. *P < 0.05

Next, the effects of PIK3CA mutation on FOLFOX chemotherapy sensitivity were assessed in primary CRC cells. Two primary cells, CC-1 and CC-2, were in vitro cultured from ascites of advanced CRC patients (Supplemental Fig. 1a). CC-1 cells were observed with *PIK3CA*-H1047R mutation, whereas CC-2 was a *PIK3CA* wild-type cells. Western blot assays showed that PI3K/Akt signaling was universally activated in CC-1 cells compared to CC-2 (Fig. 2a). The application of perifosine (an AKT inhibitor)^21^ or LY294002 (a PI3K inhibitor) markedly attenuated PI3K/Akt signaling in CC-1 cells (Fig. 2a).

Next, we tested whether PIK3CA mutation correlated with enhanced 5-FU chemotherapy resistance in primary cell lines. Our results showed higher IC50 of CC-1 cells to the treatment of 5-FU than *PIK3CA*-wild type CC-2 cells (IC50: 1.73 vs. 0.16 μM, *p* < 0.01, Fig. 2b). Importantly, when we combined perifosine or LY294002 with 5-FU, the IC50 of 5-FU treatment in CC-1 cells was significantly reduced (0.24 or 0.12 μM vs. 1.73 μM, *p* < 0.01, Fig. 2c). Western blotting assays also indicated that lower level of cleaved Caspase-3 protein expression in CC-1 than CC-2 with 1 μM 5-Fu treatment, and the combination with perifosine or LY294002 increased cleaved Caspase-3 expression (Fig. 2d). Decreased percentage of apoptosis cells in CC-1 was also showed in flow cytometry assays, whereas perifosine or LY294002 combination treatment restored the sensitivity to 5-FU (Fig. 2e and Supplemental Fig. 1b). Exogenous mutant PIK3CA expression was performed with HCT116, a colorectal cancer cell line (Supplemental Fig. 2a), which showed increased 5-FU chemotherapy resistance (Supplemental Fig. 2b). Perifosine or LY294002 treatment restored the sensitivity to 5-FU chemotherapy in HCT116-PIK3CA-H1047R cells (Supplemental Fig. 2c, d). Increased cell apoptosis was induced with perifosine or LY294002 treatment in PIK3CA-H1047R transfected cells (Supplemental Fig. 2e).

Furthermore, xenografts of CC-1 cells was planted in nude mice. Then, we treated with FOLFOX medium (25 µM 5-FU and 0.625 µM oxaliplatin) with or without perifosine. The xenografts showed significant chemotherapy resistance to FOLFOX alone, whereas significant tumor growth inhibition was observed when combined with PI3K/Akt inhibitor (Fig. 2f and Supplemental Fig. 1c, d). These data suggested that PIK3CA mutation was involved in first-line chemotherapy resistance in CRC. Moreover, PI3K/Akt signaling inhibitors can restore the efficacy of FOLFOX regimens.

### PIK3CA mutation and PI3K/Akt signaling increases CRC stem cells survival

**Fig. 3:**
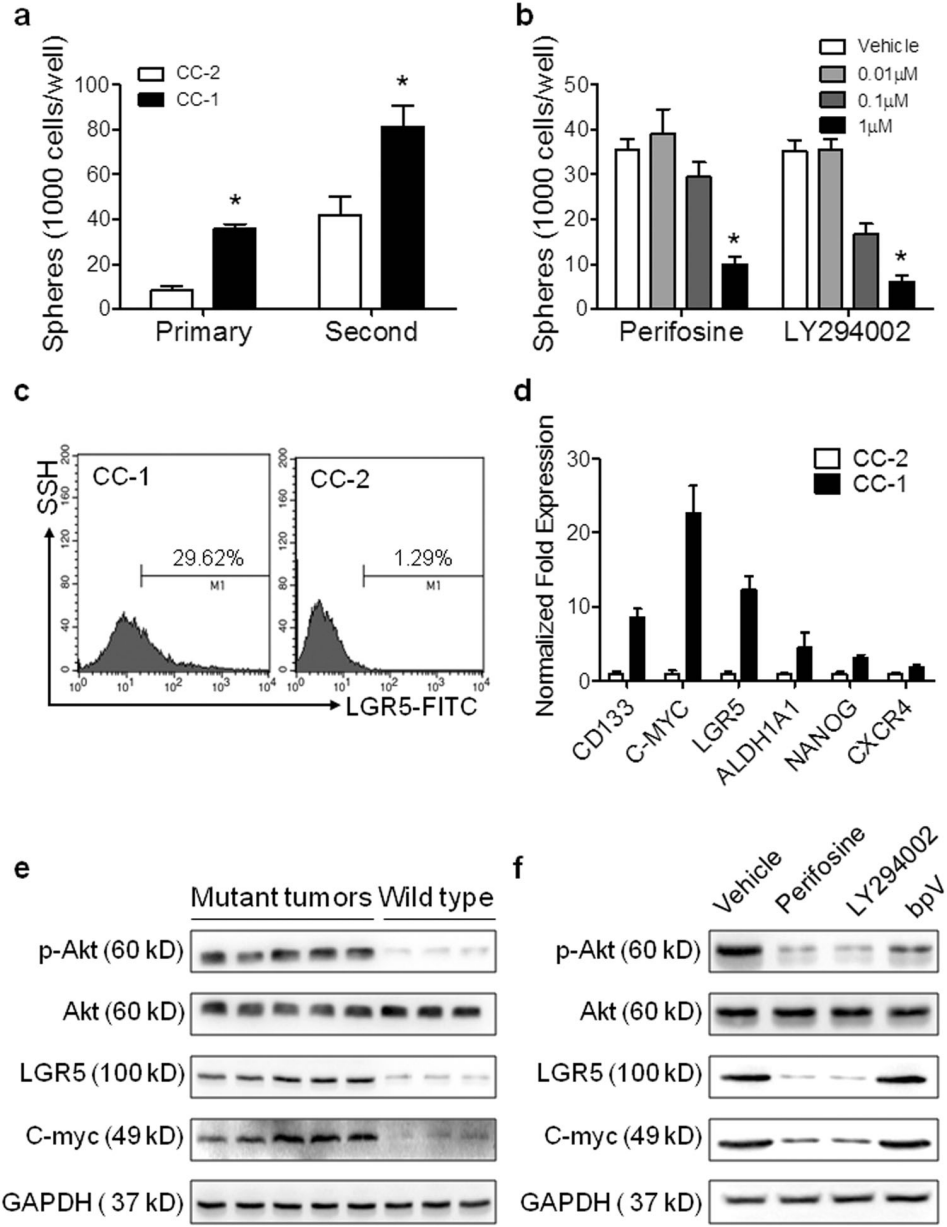
PIK3CA mutation related PI3K/Akt signaling increased CRC stem cell survival. **a** Quantitative analysis of primary and secondary sphere formation by CC-1 and CC-2 cells. **b** Quantitative analysis of sphere formation by CRC cells treated with perifosine or LY294002. **c** Flow cytometry analysis of LGR5 expression in CC-1 and CC-2 cells. **d** qRT-PCR analysis of stem cell markers, CD133, C-MYC, LGR5, ALDH1A1, NANOG and CXCR4 genes expression in CC-1 and CC-2. **e** Immunoblots showing p-Akt, Akt, LGR5, C-myc and GAPDH (loading control) expression levels in PIK3CA mutant tumor tissue comparing with that without mutation. **f** Immunoblots showing p-Akt, Akt, LGR5, C-myc and GAPDH (loading control) expression levels in CC-1 cells treated with perifosine, LY294002 or BpV (phen) comparing with vehicle

The failure of FOLFOX treatment against CRC is due to the enrichment of resistant cells, especially CRC stem cells^22^. Therefore, we further investigated the potential role of PI3K/Akt activation in CRC stem cells proliferation and survival. The PIK3CA-mutant cells, CC-1 showed increased sphere formation ability in primary and second generation of spherical cells (Fig. 3a and Supplemental Fig. 3a). Furthermore, perifosine or LY294002 treatment decreased the sphere numbers in a dose-dependent manner (Fig. 3b), which confirmed the PI3K/Akt pathway plays an important role in CRC stem cell survival. Simultaneously, higher percentage of LGR5-positive cells (CRC stem cells marker) was shown in CC-1 cells than CC-2 cells (Fig. 3c and Supplemental Fig. 3b). The qRT-PCR assays also indicated elevated expression of cancer stem cell markers in CC-1 cells than CC-2 cells (Fig. 3d).

To investigate the mechanisms of PIK3CA mutation on CRC stem cells, we determined the PI3K/Akt signaling pathway activation and CSC markers in CRC tumor tissues by western blotting. Increased pAkt levels were observed in PIK3CA mutant tumor tissues comparing with those wild type tumors (Fig. 3e). Meanwhile, the expression levels of LGR5 and c-myc were also increased in PIK3CA mutant tumor tissues comparing with wild type tumors (Fig. 3e) Further immunobloting analysis was also performed with CC-1 cells (Fig. 3f). The results showed that perifosine or LY294002 treatment inhibited PI3K/Akt signaling, and LGR5 and c-myc expression was also significantly decreased after treatment (Fig. 3f). However, treatment with bpV (phen)^23^, a PTEN inhibitor, did not cause significant decrease in LGR5 and c-myc expression (Fig. 3f). Altogether, these data suggest that PIK3CA mutation evokes CRC stem cell survival and proliferation, from which cells further resistance to chemotherapy. Moreover, PI3K/Akt signaling can be a potential target for FOLFOX-resistant CRC tumors, especially PIK3CA mutant tumors.

### Prognostic Significance of *PIK3CA* mutation and LGR5 expression

**Fig. 4:**
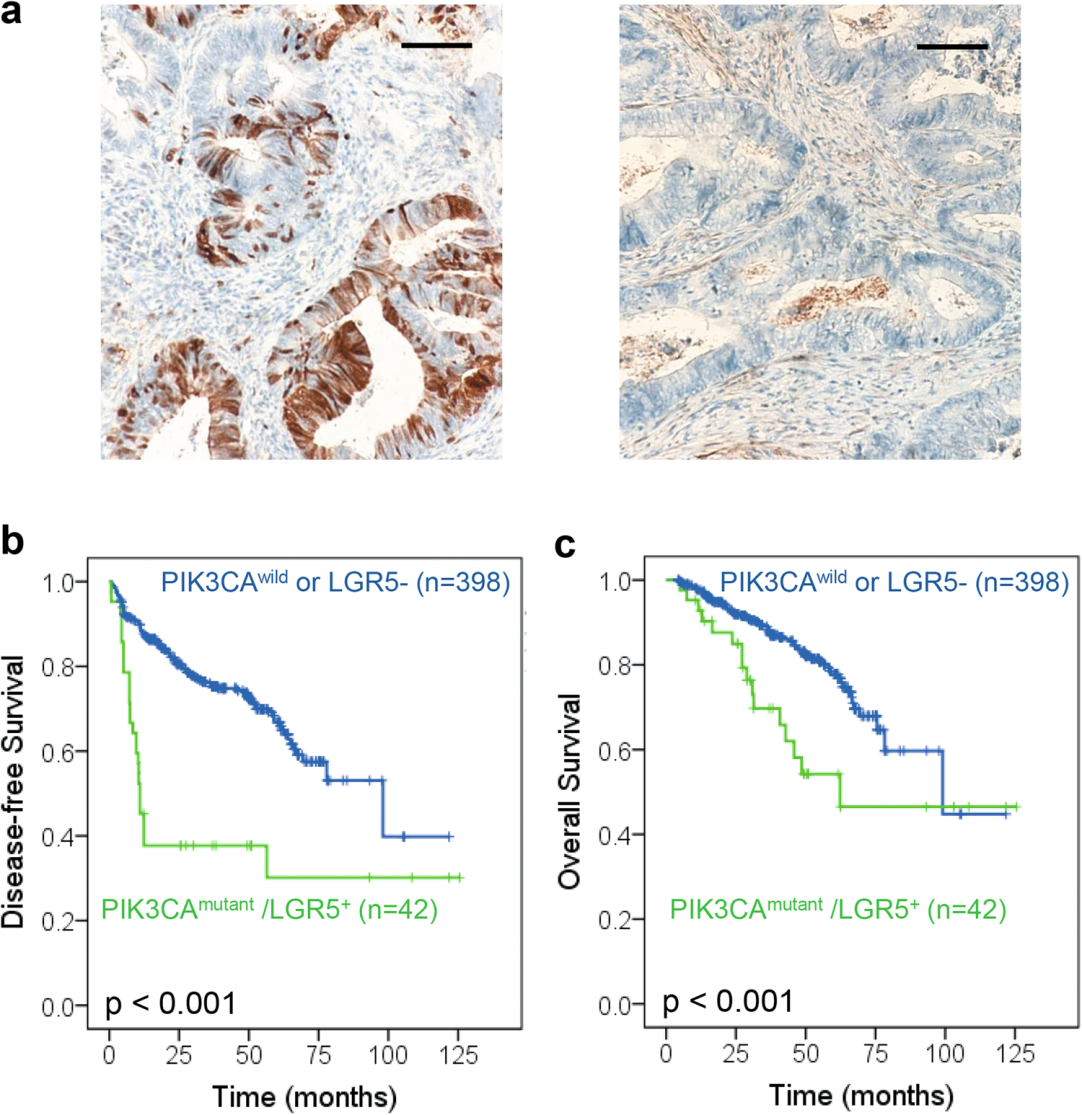
The prognostic value of PIK3CA mutation and LGR5^+^ in CRC patients. **a**, **b** Kaplan–Meier analysis of the correlation between PIK3CA mutation and disease-free survival of 440 CRC patients from Jinan Military General Hospital. **c** Kaplan–Meier analysis of the correlation between PIK3CA mutation and overall survival of 440 CRC patients

Further analysis was performed for the prognostic significance of *PIK3CA* mutation and LGR5 expression. LGR5 levels were determined with IHC staining and German semi-quantitative scores system. LGR5-positive cells showed a heterogeneity location (Fig. 4a). Totally, 34% of patients were classified into LGR5-positive group.

The median follow-up period was 40.97 (4.17–125.47) months. Totally, 84 patients (19.09%) died by the latest follow-up. The median periods of overall survival (OS) and disease-free survival (DFS) were 38.37 and 32.65 months, respectively. Kaplan–Meier analysis showed a worse prognosis in OS and DFS in *PIK3CA* ^mutant^/LGR5^+^ group than other patients (*p* < 0.001, respectively. Fig. 4b,c). Furthermore, worse prognosis was also observed in *PIK3CA*-exon 9 or exon 20 mutant groups than corresponding other patients (Supplemental Fig. 4a, b). Moreover, the prognostic value of *PIK3CA* was also validated in another cohort, which indicated poor prognosis of the patients with *PIK3CA* mutant tumor (Supplemental Fig. 4c). Univariate analysis revealed that *PIK3CA* ^mutation^/LGR5^+^ was associated with a significantly shorter DFS and OS (HR = 3.308, 95% CI: 2.158–5.070, *p* < 0.001; HR = 2.275, 95% CI: 1.310–3.950, *p* = 0.004, respectively. Table 4). Multivariate analysis was also performed with baseline prognostic variables included age, gender, ECOG, PIK3CA, histological grade, clinical stage and lymph node metastasis, which indicated *PIK3CA* ^mutation^/LGR5^+^ was a detrimental factor for both DFS and OS (HR = 3.148, 95% CI: 1.987–4.986, *p* < 0.001; HR = 2.221, 95% CI: 1.221–4.040, p = 0.009, respectively. Table 4). Moreover, our results also showed independent prognostic value of ECOG scores in both DFS and OS (HR = 2.308, 95% CI: 1.390–3.831, *p* = 0.001; HR = 2.924, 95% CI: 1.586–5.393, *p* = 0.001, respectively. Table 4).

**Table 4:** Univariate and Multivariate Analyses of Disease-Free Survival and Overall Survival according to PIK3CA mutant

Variable analysis	Disease-free survival			Overall survival		
	HR	95% CI	*p* value	HR	95% CI	*p* value
Univariate	*N* = 440			*N* = 440		
*PIK3CA*^m^/LGR5^+^	3.308	2.158–5.070	<0.001	2.275	1.310–3.950	0.004
Multivariate	*N* = 440			*N* = 440		
Age	0.942	0.672–1.321	0.729	1.011	0.650–1.574	0.960
Gender	0.792	0.553–1.135	0.204	0.795	0.501–1.259	0.327
*PIK3CA*^m^/LGR5^+^	3.148	1.987–4.986	<0.001	2.221	1.221–4.040	0.009
ECOG	2.308	1.390–3.831	0.001	2.924	1.586–5.393	0.001
Location	1.273	0.846–1.918	0.247	1.082	0.645–1.817	0.765
Grade	0.766	0.517–1.135	0.185	0.906	0.537–1.528	0.711
Stage	1.316	0.756–2.293	0.332	1.204	0.579–2.505	0.619
LN	1.345	0.809–2.235	0.253	1.316	0.684–2.532	0.411

The variables were compared in the following ways: Age, ≤59 years vs. >59 years; Gender, male vs. female; *PIK3CA*^m^/LGR5^+^, *PIK3CA*^mutant^/LGR5^+^ vs. others; ECOG, 1, 2 vs. 0; Location, RCC (cecum to the transverse colon) vs. LCRC (descending to the rectosigmoid colon and rectum Stage, III–IV vs. I–II; Grade, G3–4 vs. G1-2; LN (lymph node metastasis), metastasis vs. none

*HR* hazard ratio, *CI* confidence interval

## Discussion

Our study investigated the *PIK3CA* gene mutation and first-line chemotherapy resistance in CRC, which provide further insight in chemotherapy options. We provided evidences that *PIK3CA* mutation was correlated with lower histological grade, late clinical stage and poor prognosis. Notably, our data indicated PIK3CA mutation involved in chemotherapy resistance to first-line regimens. Sustained PI3K/Akt signaling which was induced by PIK3CA mutation enriched CRC stem cells, which contribute to chemotherapy resistance. *PIK3CA* mutation is a potential molecular biomarker for predicting and monitoring resistance to first-line chemotherapy regimens in CRC patients, which is also a potential target for chemotherapy resistant CRC tumors.

The protein product of the *PIK3CA* gene is the catalytic subunit, p110a, in class IA of PI3K^24^. Mutations of PIK3CA have been found in multiple malignant tumors, such as breast cancer^25^, endometrial cancer^26^ and bladder cancer^27^. Previous studies have reported that the frequency of PIK3CA mutation in CRC is 7%-32%^8,28–31^. Mutation hotspots are located on exons 9 and 20. Moreover, higher mutation frequency of *BRAF* and *KRAS* was also observed in *PIK3CA* mutant CRC as TCGA data and previous report^29^. In this study, exon 9 (E542K, E545K and E545D) and exon 20 (H1047R and H1047L) loci were examined via fluorescence PCR. Totally 9.55% of the CRC patients were observed with *PIK3CA* mutant tumors. Heterogeneity of PIK3CA mutations was observed among different studies^19,20^, but previous reports from China also showed slightly lower frequency of PIK3CA mutation^32,33^. Further analysis was still needed to assess the racial differences, and large cohorts study may be also necessary.

Mutation of PIK3CA leads to increased enzymatic activity of p110a and Akt signaling activation, lead to suppressed cellular apoptosis and enhanced cancer invasion^34–36^. Previous studies have supported worse prognosis in PIK3CA mutant CRC patients^37–39^. However, some previous reports showed no significant prognostic value, which was possibly correlated with different mutant site, patients’ race and ethnicity^19^. We provided further evidence with the analysis of correlation between PIK3CA mutation and clinicopathological characteristics. PIK3CA mutation was statistically correlated with late clinical stage and poor histological differentiation. Furthermore, PIK3CA mutation was a detrimental factor for CRC patients. The patients with PIK3CA mutant CRC showed significant worse prognosis. Collective data suggested a potential role of mutant PIK3CA in CRC progression^40^, which may be correlated with enhanced abnormal cell proliferation and invasive ability.

The chemotherapy of FOLFOX regimen is still the most common first-line treatment in advanced CRC according to clinical practice guidelines^41^. The regimen options are dependent on doctors’ experience, rather than evidence-based medicine. Our present study explored gene mutation biomarkers for chemotherapeutic options for better benefit. In our study, we explored PIK3CA mutation and chemotherapy response in CRC. The patients of chemotherapy resistance showed increased percentage of PIK3CA mutation. PIK3CA mutation tumors were observed with higher percentage of chemotherapy resistance, which can serve as a predicting biomarker for chemotherapy options. Therefore, multi-center double-blind randomized controlled trials with large samples are still required for further verification.

Accumulated reports supported the acquisition of stemness of cancer cells contributed to chemotherapy resistance^42–44^. Sustained PI3K/Akt signaling activation was critical for CRC stem cells enrichment^45^. Previous reports support PI3K/Akt/mTOR pathway is critical for the maintenance of colon cancer stem cells^46^. Our results have demonstrated that PIK3CA mutant induced sustain activation of PI3K/Akt signaling, from which enriched CRC stem cells. PI3K/Akt signaling inhibition attenuated the CSCs enrichment. Cancer stem cells is a sub-population of heterogeneous cancer cells, showing highly undifferentiated characteristics. LGR5 is considered as an important biomarker for CRC stem cells, which was involved in tumorigenesis and metastasis^47,48^. Given an important regulation mechanism, PI3K/Akt signaling was a potential target for eliminating CRC stem cells.

PIK3CA is a potential therapeutic target for various carcinoma treatments^33^. Idelalisib has been approved by the US Food and Drug Administration as the first PI3K inhibitor in cancer treatment^49^. There are also multiple of agents targeting PIK3CA in development^49,50^. Moreover, collective reports suggested that PIK3CA mutated CRC patients will benefit from Aspirin administration and radioembolization than wild type ones^7,51^. A detailed investigation of PIK3CA mutation in CRC progression will provide further information for improved routine practice, especially appropriate clinical use of PI3K inhibitors.

In conclusion, our work studied the PIK3CA mutation status in CRC specimens. PIK3CA mutation induced PI3K/Akt signaling contributed to the survival and proliferation of CRC stem cells, from which induced chemotherapy resistance and poor prognosis. More importantly, we indicated the potential value of PIK3CA mutation as predicting and monitoring biomarker for first-line chemotherapeutic resistance, which will contribute to better clinical regimen options for CRC.

## Materials and methods

### Patients and specimens

The retrospective study involved 440 consecutive CRC patients who were diagnosed and received surgical excision in Jinan Military General Hospital during the period from December 2006 to August 2012. The medical records were reviewed to obtain surgery information and chemotherapy regimens. All patients were administered with the first-line chemotherapy after surgical excision. The clinical data, treatment response and follow-up information of each patient were obtained by the medical records, telephone or written correspondence and death certificate. Clinicopathologic characteristics of all patients were listed in Table 1. Another CRC specimen cohort was collected from 2010 to 2017 in 401 Hospital, which was analyzed for prognostic value of PIK3CA mutation. Our study was reviewed and approved by the Ethics Committee in Jinan Military General Hospital and 401 Hospital. Criteria for treatment non-effective response were defined as recurrence, metastasis or death occurred within three years after first-line chemotherapy treatment.

### Cell lines and cell cultures

Primary CRC cells CC-1/2 were obtained from the ascites of advanced colon cancer patients. HCT116 was obtained from ATCC. Cells were cultured in Dulbecco’s modified Eagle medium (DMEM; 4.5 g/L d-glucose) (Gibco, Grand island, NY) supplemented with 10% FBS and 1% antibiotic/antimycotic in tissue culture flasks in a humidified incubator at 37 °C in a humidified incubator with 5% CO_2_. The medium was changed two times a week and cells were passaged using 0.05% trypsin/EDTA. PIK3CA-H1047R plasmid was purchased form Addgene (#14572, Cambridge, MA, USA)^52^. HCT116 cell transfection was performed as previous report^53^. In some experiments, the cells were exposed to FOLFOX (25 µM 5-FU and 0.625 µM oxaliplatin) for 72 h. Perifosine (KRX-0401, Selleck Chemicals, USA), LY 294002 (BioVision, USA), bpV (phen) (BioVision, USA) were administrated as described.

### DNA extraction and PIK3CA mutation analysis

Formalin-fixed paraffin-embedded (FFPE) materials of primary tumor tissues were collected for *PIK3CA* mutation analysis. Pathologists reviewed all FFPE specimens. Three sections, 10 µm in thickness, were prepared from each specimen. FFPE specimen DNA extraction kits-AmoyDx® DNA FFPE Tissue Kit (AmoyDx, China) were used according to the manufacturer’s protocols. The concentration and purity of extracted DNA were assessed by Nanodrop spectrophotometry (AmoyDx, China).

Mutations on *PIK3CA* exons 9 and 20 were detected with AmoyDx® PIK3CA Five Mutations Detection Kit (AmoyDx, China) with real-time PCR assays, which employed mutation-specific primers and a *PIK3CA*-targeted proprietary fluorescent probe to detect low-copy number (1%) PIK3CA-mutant DNA in tumor tissue. Mutation loci on exon 9 included E542K, E545K and E545D; mutation loci on exon 20 included H1047R and H1047L. All reactions proceeded in 20 µL volumes according to the manufacturer’s protocols with Mx3000P^TM^ real-time PCR system (Agilent, Germany). The data were analyzed with Stratagene Mxpro software. Positive results of H1047L, E545K and E545D were defined as Ct <26, while H1047R and E542K were defined as Ct <25.

### MTT assays

The survival of cells was determined by MTT (3-(4,5-Dimethylthiazol-2yl-)-2,5-diphenyl tetrazolium bromide) assay (C0009, Beyotime, China). In principle, the viable cell number is directly proportional to the purple formazan color of the reduced MTT dye, which can be quantitatively measured by spectrophotometry. Briefly, 3000 cells were plated in quadruplets in 96-well flat-bottom tissue culture plates. After treatment with compounds for certain periods as described in respective figure legends. In short, 10 μL of 10 mg/mL MTT solution was added to each well and incubated for 2 h at 37 °C. The developed color density was then measured spectrophotometrically at 570 nm using the spectrophotometer (Nanodrop, AmoyDx, China).

### Apoptosis assays

CRC cells were seeded onto six-well plates at a density of 10 × 104 cells per well and treated with perifosine, LY294002 or 5-Fu. The cells were then incubated for 48 h and stained with FITC-conjugated Annexin V and propidium iodide (PI) using an Annexin V-FITC Apoptosis Detection kit (C1063, Beyotime, China) according to the manufacturer’s recommendation. Signals of FITC-conjugated Annexin V and PI were detected using a BD LSR II flow cytometer. The percentage of apoptotic cells was determined via flow cytometry. Data was presented as the mean ± S.E.M. from three independent experiments.

### Spheroid formation assay

CRC cells were conduct with trypsin to single cell from adhere cultures. Next, CC-1/2 cells (1000 cells per well) were seeded in 24-well ultra-low attachment plates and maintained serum free DMEM/F12 medium supplemented with B27 (20 μl/ml, Life Technologies, Carlsbad, CA, USA) growth factor (EGF) (20 ng/ml, peproTech, Rocky Hill, NJ, USA), human recombinant fibroblast growth factor 2 (bFGF) (20 ng/ml, peproTech). Next day, cells were treated with or without perifosine (S1037, Selleckchem, Houston, TX, USA) or LY294002 (S1105, Selleckchem). Cells were incubated for 7 days and the number of spheroids were counted in control and treated groups under an Olympus inverted microscope with a 10 × magnification. The spheres > 100 µm were counted as tumor sphere-forming units.

### Flow cytometry analyses

For determining the CRC stem cells, cells were cultured until 60% confluence. Cells were treated with 25 µM 5-FU for 24 h with or without 20 µM perifosine or LY294002 for an additional 8 h. After treatment, cells were harvested at different time intervals, washed with ice-cold PBS and processed for FACS analysis as described previously^54^. FITC-conjugated anti-CD133 (130-104-322, Miltenyi), FITC-conjugated anti-LGR5 (130-112-508, Miltenyi). Cells were subjected to flow cytometry analyses using a BD LSR II flow cytometer. The data were analyzed and presented using FlowJo software (Tree Star, Ashland, OR, USA).

### Quantitative real-time PCR (qRT-PCR)

Total RNA was isolated from control and treated cells with RNAzol reagent (Invitogen, Carlsbad, CA, USA). The expression of mRNA levels of stemness genes were determined by qRT-PCR using Bio-Rad CFX96 Real Time System C1000 Thermal Cycler. The sequences of the specific primer for CD133, C-MYC, LGR5, ALDH1A1, NANOG, CXCR4 and GAPDH were described before^54^. The level of GAPDH mRNA was used as the internal control.

### Western blot analysis

Fresh tumor tissues were frozen with liquid nitrogen and lysed with cell lysis buffer (Cell Signaling Technologies). Western blot analysis was conducted as previously described^55^. Primary antibodies were listed as follows: rabbit anti-human Akt monoclonal antibody (mAb) (#4691, 1:1000, Cell Signaling Technology, Danvers, MA), rabbit anti-human pAkt mAb (#4060, 1:1000, Cell Signaling Technology), rabbit anti-human GAPDH mAb (#5174, 1:1000, Cell Signaling Technology), rabbit anti-human caspase-3 (#9662, 1:1000, Cell Signaling Technology), rabbit anti-human c-myc polyclonal antibody (ab19312, 1:800, Abcam, Cambridge, UK), rabbit anti-human LGR5 mAb (ab75850, 1:800, Abcam), Rabbit anti-Flag mAb (#14793 Cell Signaling Technology). Secondary antibodies were HRP-labeled goat anti-rabbit IgG (H + L) (A0208, 1:5000, Beyotime).

### Xenografts and perifosine treatment

Six-week-old male nude mice were subcutaneously injected with 1 × 10^6^ of CRC cells in each left lateral flank of mice. The mice were randomly divided into two groups when bearing about 250 mm^3^ tumors. The mice were treated with: i. PBS and 5-Fu (20 mg/kg, i.p. every day); ii. 5-Fu (20 mg/kg) and Perifosine (2.5 mg/kg, i.p. every day). Five weeks later, tumors were harvested and weighed. Tumor volume was calculated using the formula: volume = length × width^2^ × 1/2. The animal experiments were approved by the Institutional Animal Care and Use Committee of the General Hospital of Jinan Military Command PLA, in accordance with the Guide for the Care and Use of Laboratory Animals.

### Immunohistochemical staining (IHC)

IHC were performed as previous report^53^. Primary antibody against human LGR5 mAb (ab75850, Abcam) were applied for immunohistochemistry analysis. The IHC staining level was assessed with German semi-quantitative scoring system, described as previous report^53^.

### Statistical analysis

All the data were analyzed with SPSS ver. 19.0. The correlation of PIK3CA mutation and clinicalpatholical variables were assessed using Fisher’s exact test. X-tile 3.6.1 software was used to determine the optimal cut-off values. PFS and OS were estimated with Kaplan–Meier analysis with log-rank test. Cox proportional hazards model was sued for univariate/multivariate analysis. Hazard ratios and their corresponding 95% confidence intervals were computed to provide quantitative information. All experiments were repeated at least three times and results were expressed as mean ± SEM. A *P* value of <0.05 was considered statistically significant.

## Electronic supplementary material

Supplemental material
