# Supplementary material for: PIK3CA mutations confer resistance to first-line chemotherapy in colorectal cancer
Source: Cell Death Dis. 2018 Jul 3;9(7):739. doi: 10.1038/s41419-018-0776-6 (PMC6030128; doi:10.1038/s41419-018-0776-6)
Supplement: Supplementary file 1 — Supplemental material [file 41419_2018_776_MOESM1_ESM.docx]

**Supplemental Figures**

**
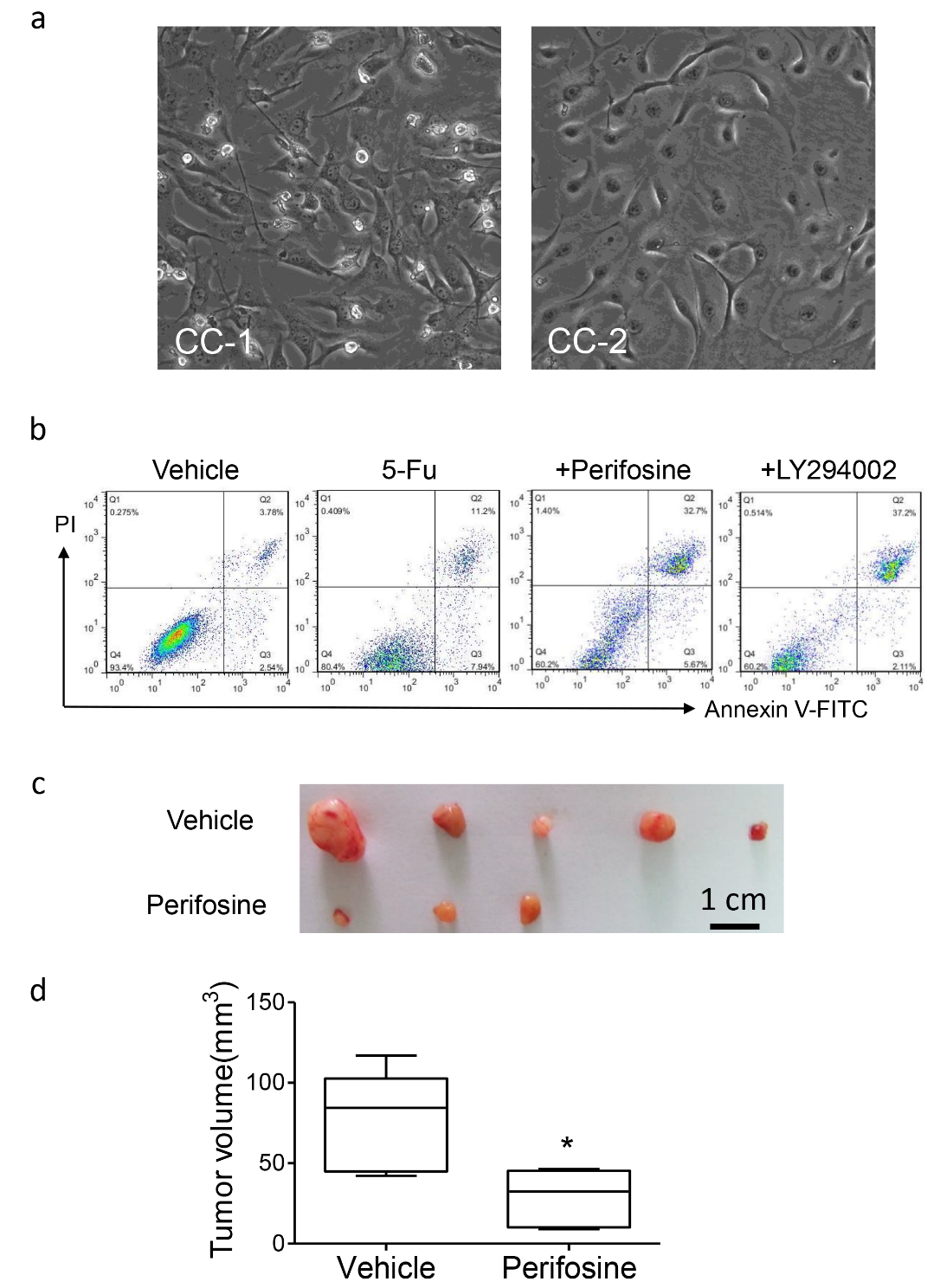
**

**Supplemental Figure 1** PIK3CA mutation correlated with first-line chemotherapy resistance.

(A) The in vitra cultured primary cells of CRC, CC-1/2. (B) Reprentative results of flowcytometry assays for apoptostic cells in different treatent as shown in Figure 2E. (C) The representative images of CRC xenograft tumors treated with or without Perifosine. (D) Quantitative analysis of the volume of the indicated xenografts.





**Supplemental Figure** 2 Exogenous mutant PIK3CA expression induces chemotherapy resistance.

(A) Western blot analysis confirmed PIK3CA-H1047R expression in transfected HCT116 cells. (B) Cell viability of transfected HCT116 cells were examined with various concentration of 5-FU treatment. HCT116-PIK3CA-H1047R cells showed higher IC50 values for 5-FU. (C) Western blot analysis of pAkt, Akt and GAPDH (loading control) in transfected HCT116 cells which were treated with perifosine or LY294002. (D) Transfected HCT116 cells were treated with perifosine or LY294002 combined with various concentration of 5-FU. IC50 values of different groups were determined. (E) Western blot analysis was performed for Cleaved casepase and GAPDH (loading control) in transfected HCT116 cells which were treated with perifosine or LY294002 combined with 5-Fu (1 μM) for 24 h.


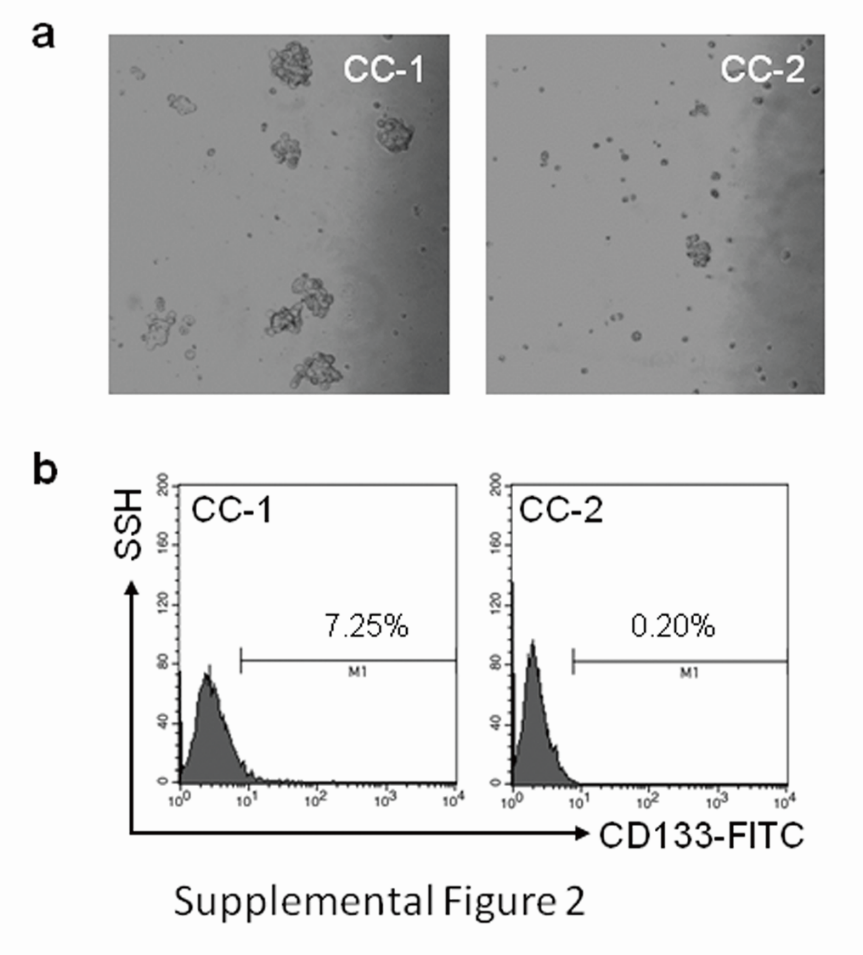


**Supplemental Figure 3** PIK3CA mutation related PI3K/Akt signaling increased CRC stem cell survival

(A) The representative images of tumorsphere derived from CRC cells(CC-1 and CC-2). (B) Representative FACS analyses of CD133 in CC-1 and CC-2, respectively. All experiments were performed independently for three times.

**
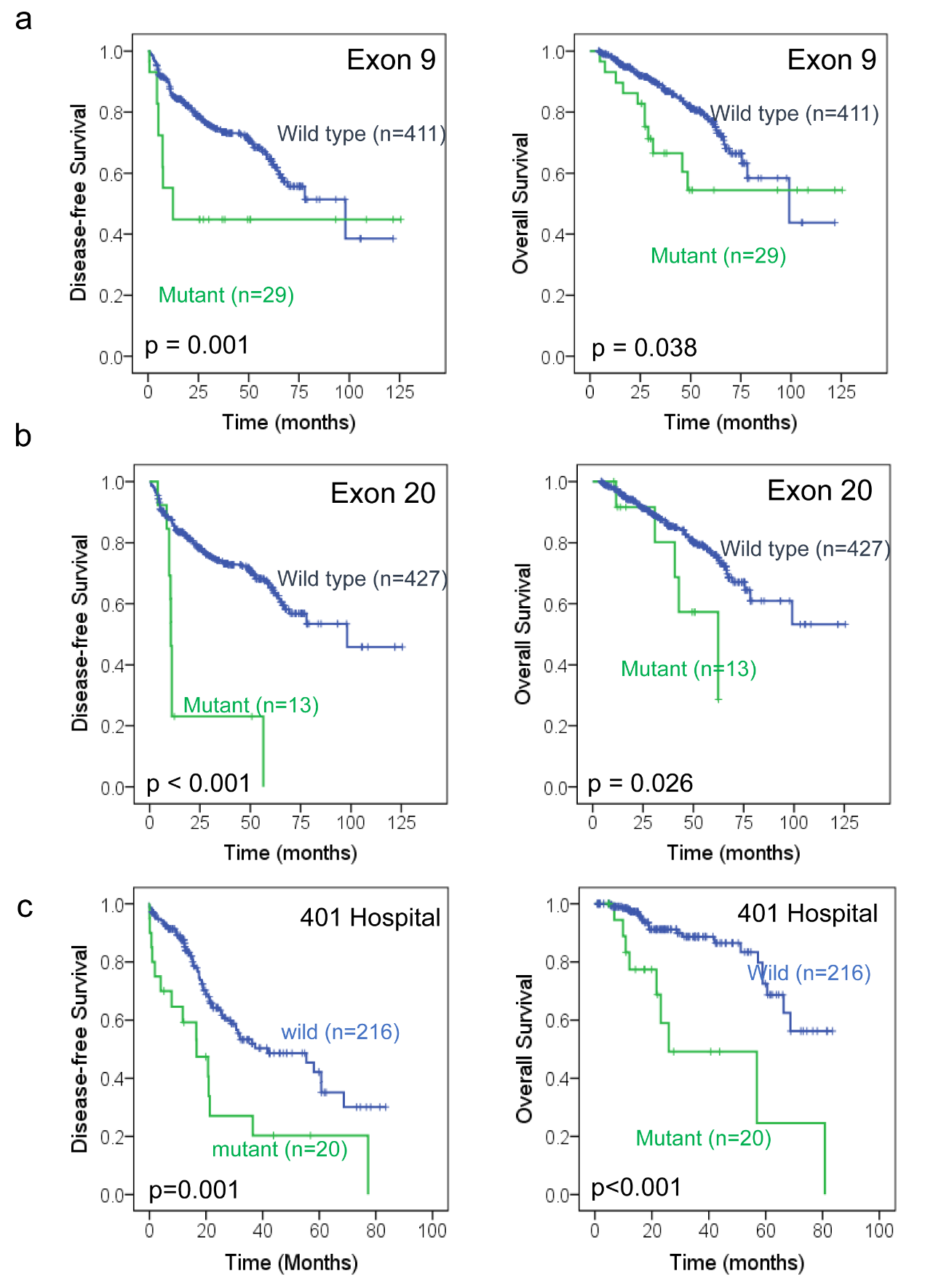
**

**Supplemental Figure 4** The prognostic value of PIK3CA mutation in CRC patients

(A) Kaplan-Meier analysis of PIK3CA mutation on exons 9 in disease-free survival and overall survival of 440 CRC patients. (B) Kaplan-Meier analysis of PIK3CA mutation on exons 20 in disease-free survival and overall survival of 440 CRC patients. (C) Kaplan-Meier analysis of the correlation between PIK3CA mutation and disease-free survival and overall survival of 236 CRC patients from Cohort 401 Hospital.
